# MyotonPRO Is Not Comparable to Shear Wave Elastography in the Measurement of Rectus Femoris Muscle Stiffness due to Interference of Subcutaneous Adipose Tissue

**DOI:** 10.1111/sms.70095

**Published:** 2025-07-25

**Authors:** Cameron D. Ley, Eduardo Martinez Valdes, Conall F. Murtagh, Jonathan Power, Lee Nobes, Barry Drust

**Affiliations:** ^1^ School of Sport, Exercise and Rehabilitation Sciences University of Birmingham Birmingham UK; ^2^ Sports Science Department Liverpool Football Club and Athletics Ground Ltd Liverpool UK; ^3^ Medical Department Liverpool Football Club Liverpool UK

**Keywords:** Biomechanics, Muscle, MyotonPRO, Shear wave elastography, Stiffness

## Abstract

Tissue biomechanical properties are important for optimizing musculoskeletal performance and reducing injury risk. The MyotonPRO was developed as a simple, portable, noninvasive, and highly reliable method for the estimation of muscle stiffness. It has not, however, been comprehensively validated against shear wave elastography (SWE), a validated ultrasound‐based method for assessing tissue stiffness. To assess this, 20 participants completed two visits in which rectus femoris (RF) stiffness was measured twice with the MyotonPRO device and SWE at three distinct muscle regions (proximal, medial, and distal), three muscle lengths (relaxed—REL, neutral—NEU, and passively stretched—PAST), and four depths (skin—SKIN, fascia—FAS, superficial muscle—SUP, and deep muscle—DEEP). Additionally, subcutaneous adipose tissue thickness (SAT) was recorded at each location under every condition using B‐mode ultrasound imaging. When pooling the regions in REL, the MyotonPRO exhibited a weak significant negative correlation with SWE in DEEP (*r* = −0.27, *p* = 0.020), but not at SKIN, FAS, or SUP. In NEU and PAST, stiffness estimated by the MyotonPRO had a moderate positive relationship with SWE in the SKIN (*r* = 0.66, *p* < 0.001; *r* = 0.60, *p* < 0.001), but weaker relationships with deeper tissue (FAS, *r* = 0.26, *p* = 0.023; *r* = 0.20, *p* = 0.069; SUP, *r* = 0.24, *p* = 0.036; *r* = 0.26, *p* = 0.023; DEEP, *r* = 0.315, *p* = 0.008; *r* = 0.454, *p* < 0.001, respectively). Stiffness measured by the MyotonPRO exhibited significant moderate‐to‐strong negative correlations with SAT in every region under every condition, except for medially in REL. These results suggest that the two methods cannot be used interchangeably to estimate the stiffness of the RF muscle.

## 1 Introduction

Stiffness is a mechanical property of biological tissue quantified by the extent of tissue deformation (strain) in response to an applied force (stress) [1]. It is calculated as the slope of a material's stress/strain curve [2]. Changes in muscle stiffness can impact athletic performance, with higher levels of stiffness associated with improved performance in explosive actions such as sprinting and jumping, but also with a greater risk of injury [3, 4, 5, 6]. Measurement of muscle stiffness is also relevant in clinical settings, with augmented muscle stiffness aligning with muscle contractures and rigidity observed in patients with cerebral palsy and Parkinson's disease [7, 8, 9]. Tensile testing is the gold standard for assessing material stiffness. In muscle, this method involves dissecting the muscle, clamping one tendon, applying increasing force to the other tendon, and measuring the resulting change in muscle length [10, 11]. However, this approach has clear limitations when applied to athletic and clinical populations, necessitating the development and validation of non‐invasive techniques for estimating tissue stiffness.

Shear wave elastography (SWE) is an ultrasound‐based technique in which shear waves are induced in muscle, with the speed of their propagation through the tissue tracked by the transducer. A faster shear wave velocity (SWV) indicates greater tissue stiffness [12]. This technique has already been implemented in homogenous, isotropic tissue in clinical settings, with the quantification of stiffness being utilized in the diagnosis of liver cirrhosis and identification of cancerous breast masses [13, 14]. SWE has been correlated with tensile testing of muscle tissue in cadavers, validating its use in nonhomogenous, anisotropic tissue [10, 11]. Musculoskeletal SWE has been performed in clinical research settings, detecting muscular differences between children with cerebral palsy and healthy age‐matched controls [7]. It has also been applied in athletic settings, assessing changes in muscle stiffness following damaging exercise [15, 16]. SWE is therefore considered a valid method for the noninvasive estimation of muscle stiffness, with applications in clinical and athletic research settings.

The MyotonPRO is a novel handheld device developed as a portable method for assessing muscle mechanical properties as a simpler alternative to SWE. This is because SWE is expensive, requires more extensive operator training, and is not easily portable. The MyotonPRO estimates dynamic stiffness based on tissue deformation and oscillation in response to the application of a mechanical force at the skin [17]. Myoton define dynamic stiffness as the resistance of biological soft tissues to a force of deformation [18]. Research has compared the MyotonPRO with SWE in muscles and gel phantom models, finding significant positive correlations between the two [17, 19, 20]. This provides some evidence validating the MyotonPRO, indicating that it may be a viable alternative to SWE. Many of these correlations, however, do not indicate a strong relationship between the two methods, justifying the need to test different examination conditions to optimize agreement between the devices by manipulating muscle length. With regard to the RF muscle, Lee and colleagues estimated stiffness with participants in the supine position, finding a significant Pearson's correlation of 0.42 between the devices' measurements [20]. Moreover, it may also have clinical relevance, as research has shown an association with rigidity in Parkinson's disease [9], and could be potentially useful in athletic settings, as it has proven to be sensitive to the eccentric exercise‐induced increase in muscle stiffness [21].

Despite this evidence, there is a known dampening effect of the mechanical impulse caused by interference of non‐target biological soft tissue with MyotonPRO use. Significant negative correlations have been found between dynamic stiffness of the RF and vastus lateralis (VL) muscles, measured by the MyotonPRO, and subcutaneous adipose tissue thickness (SAT) measured by skinfold calipers and ultrasound, respectively [22, 23]. Consequently, further research is required to establish the efficacy of the MyotonPRO in estimating muscle stiffness. Therefore, the aim of this study was to examine the relationship between stiffness of the rectus femoris (RF) muscle and its supra‐adjacent tissue estimated by SWE and the MyotonPRO under different muscle lengths. We chose to assess the RF muscle as its biarticular anatomy means it contributes to both hip flexion and knee extension, giving it a high functional significance in movement and exercise. We also attempted to assess the impact of SAT on dynamic stiffness values recorded by the MyotonPRO. Muscle length was manipulated to alter tissue stiffness and determine the sensitivity of the MyotonPRO to these changes, to which SWE is sensitive.

## 2 Materials and Methods

### 2.1 Participants

A total of 20 healthy active participants (10 males and 10 females; age: 26.3 ± 4.0 years; height: 1.70 ± 0.10 m; weight: 67.7 ± 12.7 kg; BMI: 23.2 ± 2.8 kg/m [2]) volunteered for this study. Participants had no history of musculoskeletal injury in the examined limb. Ethical approval for the study was obtained from the Science, Technology, Engineering, and Mathematics Committee at the University of Birmingham (ERN_0432‐Apr2023) and all procedures conformed to the Declaration of Helsinki. Participants provided written informed consent prior to measurements. Sample size was determined via a power analysis (G* Power software) based on previous research, with an effect size of 0.50, statistical power set at 0.80, and the alpha level set at 0.05 [24].

### 2.2 Experimental Design

Participants attended a familiarization session to complete eligibility screening and become accustomed to the laboratory setting and measurements which would be taken at experimental visits. Two experimental visits were conducted on separate days within a 7‐day period. Both experimental visits were completed at the same time of day to avoid effects of routine and circadian rhythm on measurements. Participants were asked to refrain from lower body exercise and consumption of alcohol and recreational drugs 24 h prior to each experimental visit. Measurements were repeated twice per experimental session, with a 30 min rest period between measurements, during which participants remained seated. This allowed the calculation of both intra‐ and inter‐day repeatability of measurements.

### 2.3 Experimental Procedures

Before starting the experiment, participant height (SECA, Hamburg Germany) and weight (Ohaus ChampII, New Jersey USA) were measured, following which participants rested in the laboratory, set at a temperature of 21°C, for 10 min on the examination table to stabilize body conditions, as per previous research [25, 26]. Muscle stiffness was estimated at three sites along the rectus femoris (RF) muscle: proximal (PROX), medial (MED), and distal (DIST). These sites were 15%, 50%, and 85% of muscle length while the muscle was relaxed with participants supine (REL), respectively. Muscle length was determined using B‐mode ultrasound (US) with a 16‐linear array probe (50 mm, 4–15 MHz) (LOGIQ S8 GE Healthcare, Milwaukee, USA) to identify the proximal and distal musculotendinous junctions of the RF, which were marked on the skin using a permanent marker, and muscle length (cm) measured using a measuring tape. At these three sites, muscle stiffness was estimated in three different conditions to manipulate muscle length: relaxed, neutral, and passively stretched. In the relaxed (REL) condition, participants laid supine on an examination table. In the neutral (NEU) condition, participants were seated in an isokinetic dynamometer (IKD) (Biodex System 3 Dynamometer, Biodex Medical Systems) with the hip joint fixed at 110° relative to full hip flexion and the knee joint fixed at 90° of flexion. In the passively stretched (PAST) condition, participants were laid down in the IKD, with the hip joint fixed at 155° relative to full hip flexion and the knee joint fixed at 90°. To ensure the consistency of measurement location between measurements, the location of the US and MyotonPRO probes were marked on the skin in permanent marker, while participants were supine and anatomical landmarks were identified using B‐mode US. During measurements, participants were asked to remain still and relaxed, avoiding muscle contractions. The dominant leg, defined as the preferred leg used to kick a football, was selected for stiffness measurement. SAT was determined using the measuring tool on the US device and was measured from the center of the image, corresponding with the location at which MyotonPRO dynamic stiffness measurements were taken.

### 2.4 Shear Wave Elastography

**FIGURE 1:**
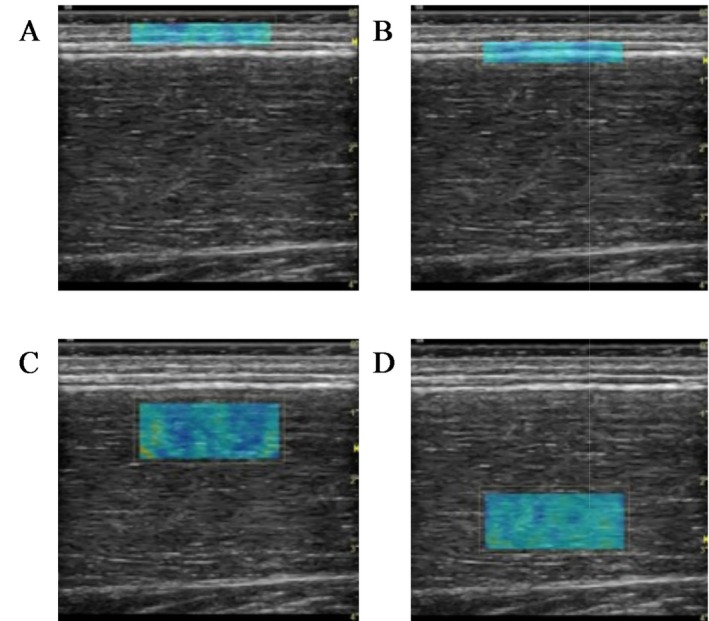
Example images of the regions of interest used to estimate (A) skin, (B) fascia, (C) superficial muscle, and (D) deep muscle stiffness using shear wave elastography.

Stiffness was estimated via SWE using an ultrasound device equipped with SWE on a 9‐linear array probe (44 mm, 2–8 MHz) (LOGIQ S8 GE Healthcare, Milwaukee, USA) which was positioned longitudinally to the muscle fascicles. The US SWE system has an upper limit for SWV measurement of 15 m/s. All SWV measurements were performed by a PhD student with 1 year of MSK ultrasonography and SWE experience. At each measurement site (PROX, MED, and DIST), SWE was measured at four different tissue sites: skin (SKIN), deep fascia (FAS), superficial muscle (SUP), and deep muscle (DEEP). For measurements of SKIN, FAS, and DIST SUP and DEEP, a 2.2 × 0.5 cm region of interest (ROI) was selected. For PROX and MED SUP and DEEP, a 2.2 × 1.0 cm ROI was selected (Figure 1). Depth of ROI positioning varied between participants based on the thickness of SAT, and other overlying fibrous structures. This ROI displayed a real‐time shear wave elastogram color map, which was recorded for 15–20s. The three most visually similar consecutive frames were used for image processing, wherein mean SWV (m/s) was determined. The mean values from these three consecutive images were used for further analysis. During measurements, a probe holder (Manfrotto, Italy) was used to ensure stability of the transducer during measurements and avoid compression of the tissue below. Furthermore, to avoid compression, a thick layer of acoustic coupling gel (> 1 mm) was used, ensuring it could be visualized on the B‐mode image. If there were voids or saturation in the shear wave elastogram, the ROI and transducer positions were adjusted to optimize image quality and consistency. In instances where voids could not be avoided, frames with the least amount of saturation were selected for analysis.

### 2.5 Myotonometry

A MyotonPRO (Myoton AS, Tallinn, Estonia) device was used to quantify muscle tissue dynamic stiffness. Measurements with the MyotonPRO were taken at the same sites as with SWE, from the center of the box drawn around the US transducer. The device was held with two hands, with the arms resting on the examination table or IKD to ensure maximum stability and minimize movement of the device. The MyotonPRO recorded three measurements at each site, from which the mean was used for analysis. As per the user manual, the MyotonPRO applies a precise mechanical compression force of 0.6 N for 15 ms, inducing a decaying natural oscillation within the displaced muscle tissue which is measured by the device and used to calculate biomechanical properties of tissue.

### 2.6 Statistical Analysis

All statistical analysis was conducted using IBM SPSS software (version 29). Intra‐ and inter‐day repeatability of SWE measurements was calculated for all three muscle regions (PROX, MED, and DIST) at all four ROI depths (SKIN, FAS, SUP, and DEEP) in all three conditions (REL, NEU, and PAST), as well as for the MyotonPRO, in all three regions and conditions, using intraclass correlation coefficients (ICC). It is generally accepted that ICC values less than 0.50 indicate poor reliability, values between 0.50 and 0.75 are indicative of moderate reliability, values between 0.75 and 0.90 indicate good reliability, and values above 0.90 represent excellent reliability [27]. Normality of data was assumed following the Shapiro–Wilk test. Simple linear regressions with Pearson's correlations were performed to assess the level of agreement between stiffness measured by the MyotonPRO and at the four ROI depths with SWE, repeated for each condition. These analyses were conducted at each region separately, as well as with the regions pooled under each condition. Simple linear regressions with Pearson's correlations were also performed to assess the relationship between SAT and stiffness measured by the MyotonPRO individually at each region under each condition. For these measurements, data were excluded where SAT exceeded 2 cm because Myoton states in the user manual that the device is unable to measure tissue covered by > 2 cm adipose tissue [28]. This involved exclusion of measurements from PROX in only one participant. Strength of Pearson's correlations is reported as negligible (0.00–0.09), weak (0.10–0.39), moderate (0.40–0.69), strong (0.70–0.89), and very strong (0.90–1.00) [29]. To determine regional differences in SAT, a 3 condition (REL, NEU, PAST) by 3 location (PROX, MED, DIST) two‐way repeated measures analysis of variance (ANOVA) was performed. Post hoc pairwise comparisons were conducted using the Bonferroni correction to investigate significant differences in detail. All values reported in text and graphs are mean ± SD unless otherwise specified. For all analyses, statistical significance was set at *α* < 0.05.

## 3 Results

### 3.1 Repeatability of Shear Wave Elastography and the MyotonPRO

SWV exhibited moderate intra‐session repeatability (ICC 0.69) and good inter‐day repeatability (ICC 0.80). The MyotonPRO exhibited excellent intra‐session repeatability (ICC 0.97) and inter‐day repeatability (ICC 0.97). Full reliability values are presented in Table S1.

### 3.2 Relationship Between Shear Wave Elastography and the MyotonPRO

**FIGURE 2:**
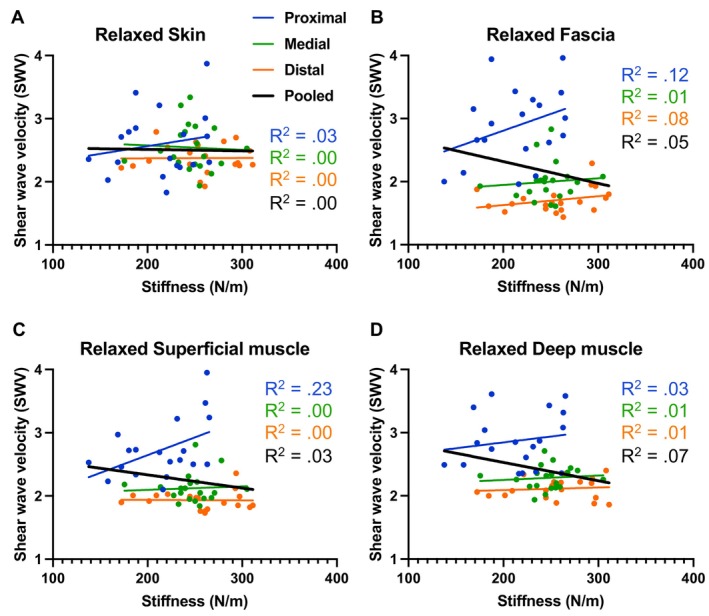
Relationship between shear wave velocity, measured by shear wave elastography at the (A) skin, (B) fascia, (C) superficial muscle, and (D) deep muscle and dynamic stiffness, measured by the MyotonPRO, including the simple linear regression *R* ^2^ values for each region separately and pooled, in the relaxed condition.

With regard to the pooled data, in REL, Pearson's correlation and simple linear regressions revealed a weak significant negative correlation between stiffness estimated by the MyotonPRO and SWV at DEEP (*r* = −0.27, *p* = 0.020, *R* ^2^ = 0.07), but no significant correlations were observed at SKIN (*r* = −0.23, *p* = 0.430, *R* ^2^ = 0.00), FAS (*r* = −0.21, *p* = 0.052, *R* ^2^ = 0.05), or SUP (*r* = −0.18, *p* = 0.084, *R* ^2^ = 0.03) (Figure 2).

**FIGURE 3:**
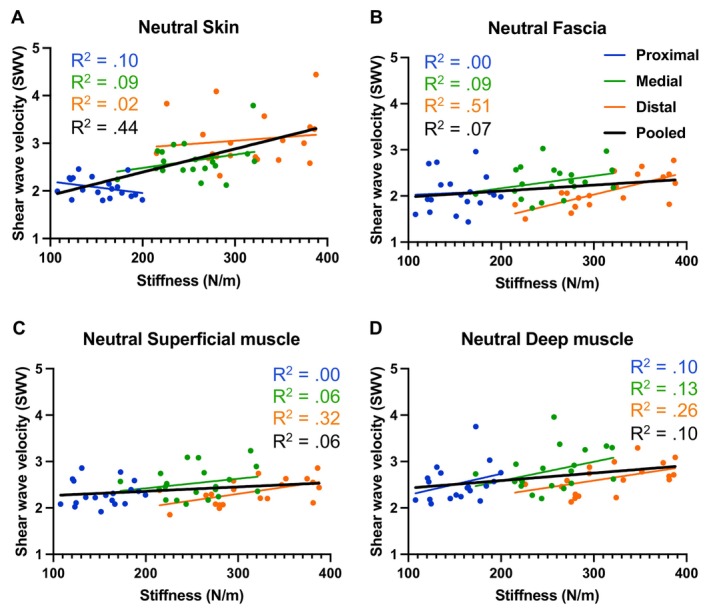
Relationship between shear wave velocity, measured by shear wave elastography at the (A) skin, (B) fascia, (C) superficial muscle, and (D) deep muscle and dynamic stiffness, measured by the MyotonPRO, including the simple linear regression *R* ^2^ values for each region separately and pooled, in the neutral condition.

Pearson's correlation and simple linear regressions of pooled data in NEU, however, reveal significant positive correlations between stiffness estimated by the MyotonPRO and SWV at all measurement depths (Figure 3). A moderate correlation was found at the SKIN (*r* = 0.66, *p* < 0.001, *R* ^2^ = 0.44), but weak correlations were observed at FAS (*r* = 0.26, *p* = 0.023, *R* ^2^ = 0.07), SUP (*r* = 0.24, *p* = 0.036, *R* ^2^ = 0.06), and DEEP (*r* = 0.32, *p* = 0.008, *R* ^2^ = 0.10).

**FIGURE 4:**
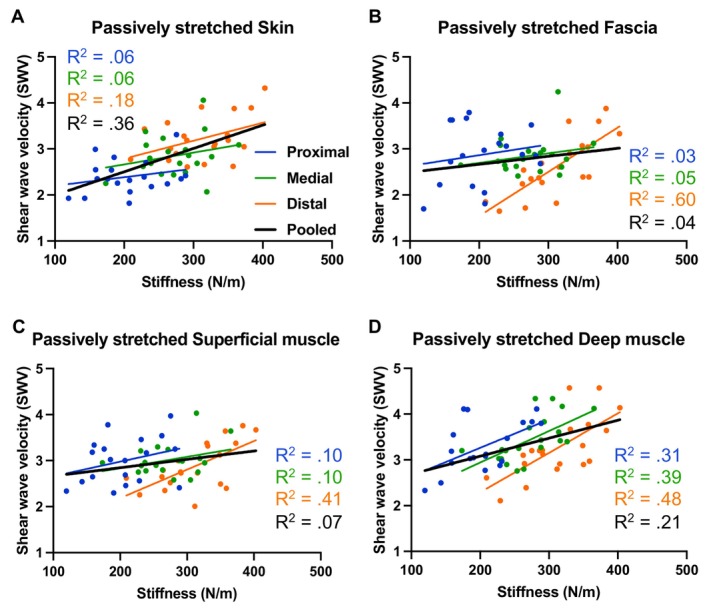
Relationship between shear wave velocity, measured by shear wave elastography at the (A) skin, (B) fascia, (C) superficial muscle, and (D) deep muscle and dynamic stiffness, measured by the MyotonPRO, including the simple linear regression *R* ^2^ values for each region separately and pooled, in the passively stretched condition.

For pooled data in PAST, significant moderate positive correlations between stiffness estimated by the MyotonPRO and SWV were observed at SKIN (*r* = 0.60, *p* < 0.001, *R* ^2^ = 0.36) and DEEP (*r* = 0.45, *p* < 0.001, *R* ^2^ = 0.21), a significant weak correlation at SUP (*r* = 0.26, *p* = 0.023, *R* ^2^ = 0.07), but no significant relationship at FAS (*r* = 0.20, *p* = 0.069, *R* ^2^ = 0.04) (Figure 4). Full data are provided in Table S2.

### 3.3 Relationship Between the MyotonPRO and Subcutaneous Adipose Tissue Thickness

**FIGURE 5:**
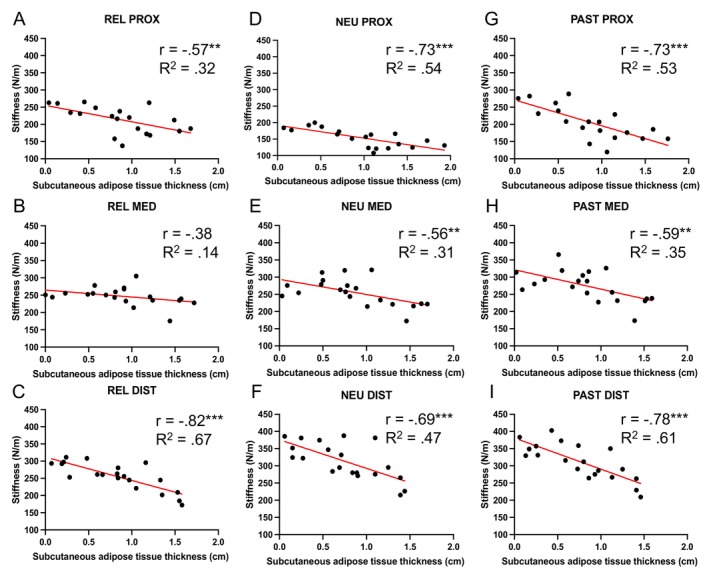
Relationship between dynamic stiffness, measured by the MyotonPRO, and subcutaneous adipose tissue thickness, measured using B‐mode ultrasound, including Pearson's *r* value and the simple linear regression *R* ^2^ value. (A–C) relaxed condition, in order of proximal, medial, and distal regions. (D–F) neutral condition, in order of proximal, medial, and distal regions. (G–I) passively stretched condition, in order of proximal, medial, and distal regions. **p < 0.010, ***p < 0.001.

Figure 5 plots dynamic stiffness against SAT under all three conditions (REL, NEU, and PAST), and at all three regions (PROX, MED, and DIST). The data show consistent moderate–strong negative correlations between dynamic stiffness and SAT which, with the exception of the weak relationship at REL MED, are statistically significant in all conditions and locations. This implies an interference effect of the less stiff SAT on the ability of the MyotonPRO to accurately quantify mechanical properties of underlying muscle.

In REL, Pearson's correlations and simple linear regressions revealed a significant moderate negative correlation between dynamic stiffness and SAT at PROX (*r* = −0.57, *p* = 0.006), with 32.34% of variance in stiffness explained by SAT, and a strong correlation at DIST (*r* = −0.82, *p* < 0.001), with 66.46% of variance in stiffness explained by SAT, but a weak, nonsignificant correlation in MED (*r* = −0.38, *p* = 0.050), where 14.30% of variance in stiffness is explained by SAT (Figure 5A–C).

In NEU, Pearson's correlations and simple linear regressions revealed a strong significant negative correlation between dynamic stiffness and SAT at PROX (*r* = −0.73, *p* < 0.001), with 53.51% of variance in stiffness explained by SAT, and moderate correlations at MED (*r* = −0.56, *p* = 0.005), with 31.03% of variance in stiffness explained by SAT, and DIST (*r* = −0.69, *p* < 0.001), with 47.29% of variance in stiffness explained by SAT (Figure 5D–F).

Similarly, in PAST, Pearson's correlations and simple linear regressions revealed strong significant negative correlations between dynamic stiffness and SAT at PROX (*r* = −0.73, *p* < 0.001), with 53.01% of variance in stiffness explained by SAT, and DIST (*r* = −0.78, *p* < 0.001), with 60.75% of variance in stiffness explained by SAT, and a moderate correlation at MED (*r* = −0.59, *p* = 0.003), with 35.00% of variance in stiffness explained by SAT (Figure 5G–I).

### 3.4 Regional Differences in Subcutaneous Adipose Tissue Thickness

**FIGURE 6:**
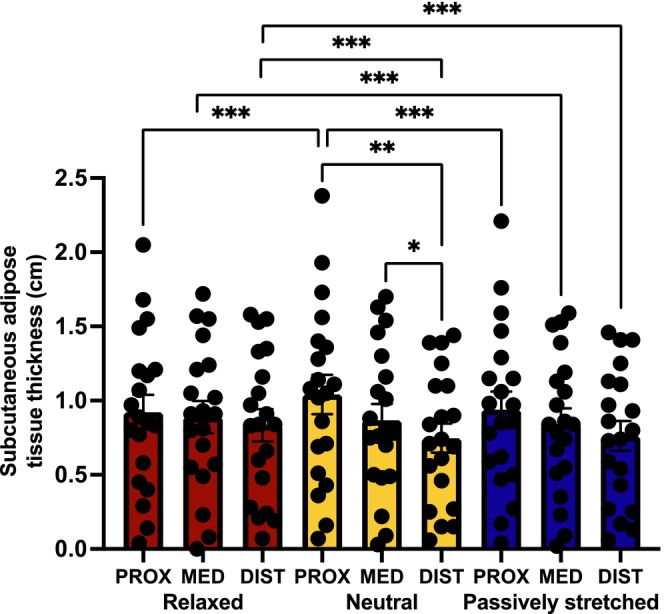
Differences between subcutaneous adipose tissue thickness measured at proximal, medial, and distal locations in the relaxed, neutral, and passively stretched conditions by B‐mode ultrasound. *p < 0.050, **p < 0.010, ***p < 0.001.

In both REL and PAST, there were no significant differences in SAT between regions (Figure 6). In NEU, however, SAT was lower at DIST than PROX (*p* = 0.003) and MED (*p* = 0.017). When comparing regions across conditions, there are further differences. At PROX, SAT is greater in NEU than in REL or PAST (both *p* < 0.001). At MED, SAT is higher in REL than in PAST (*p* < 0.001). At DIST, SAT is larger in REL than in NEU and PAST (both *p* < 0.001). These results show that localized flexion causes compression which increases SAT.

## 4 Discussion

The aims of the study were to examine the relationship between stiffness of the RF muscle and its supra‐adjacent tissue estimated by SWE and the MyotonPRO under different muscle lengths and to assess the impact of SAT on dynamic stiffness values recorded by the MyotonPRO. The present study has three main findings: (1) RF stiffness reported by the MyotonPRO has no agreement with SWV in a supine position, (2) stiffness reported by the MyotonPRO has stronger correlations with SWV of superficial tissue than of deeper tissue, and (3) stiffness reported by the MyotonPRO negatively correlates with SAT regardless of location or condition. Overall, our results show that the ability of the MyotonPRO to estimate RF muscle stiffness is limited by the interference of overlying non‐contractile tissue and is not an effective method of assessing mechanical properties in deep structures. Combined, these findings indicate that the MyotonPRO is not an effective alternative method to SWE of assessing RF muscle stiffness. This may be a function of the devices measuring different tissue properties. Since there is a stronger agreement between the two devices at longer muscle lengths, and stiffness reported by the MyotonPRO is negatively related to SAT, use of the MyotonPRO is recommended with the muscle in a stretched state, and in muscles covered by minimal overlying noncontractile tissue if the target is to optimize agreement with SWE.

In REL, there existed no positive correlation between the MyotonPRO and SWV at any location. This is particularly relevant, since measurements of the quadriceps muscles using the MyotonPRO are recommended to be taken with participants in a supine position. These findings support results observed at the VL muscle in a supine condition, where there was no relationship between stiffness assessed by the MyotonPRO and shear modulus estimated by the same model of US device as in the present study [23]. In fact, in DEEP, we found a significant weak negative correlation between stiffness estimations by SWE and the MyotonPRO. These findings are likely because SWE is sensitive to stretch‐induced changes in proximal RF stiffness caused by hip extension whilst supine. Meanwhile, estimates by the MyotonPRO are dampened by the slightly greater SAT proximally, creating a negative relationship between the two measurements. These results would seem to indicate that the guidance for participant positioning for estimating quadriceps muscle stiffness with the MyotonPRO should not be supine. Alternatively, since there is no agreement between SWE and the MyotonPRO in REL, our results may imply that the devices are measuring two distinct mechanical properties. These two hypotheses based on our results will be discussed below.

We investigated the effect of altering participant positioning (to increase muscle length) on the agreement between SWE and the MyotonPRO. Under these conditions of knee flexion in NEU and PAST, we observed significant weak–moderate positive correlations in the estimation of tissue stiffness between SWE and the MyotonPRO. However, our results reveal that these correlations are stronger at the SKIN than at deeper tissues. This would support a known limitation of the MyotonPRO, which is its limited depth of measurement. This may be a result of its mechanism of assessment in which a mechanical compression force is applied by a probe at the skin surface and the measurement of the oscillation of the displaced tissue below. If this mechanism is accurate, it would suggest that a greater preload compression force may enable a more effective assessment of deeper structures, including muscle tissue. This is supported by findings presented in Figure 5 that show negative relationships between stiffness measured by the MyotonPRO and SAT regardless of measurement location or position. Furthermore, Figure 6 highlights that there is greater SAT in proximal regions and in REL and NEU than in PAST. These findings support the hypothesis that SAT is a limiting factor on the estimation of muscle stiffness using the MyotonPRO, since better relationships are observed between the two devices in DIST and PAST, where SAT is lower. This is likely a consequence of the dampening effect on mechanical wave transmission caused by the interference of the softer adipose tissue overlying the muscle. This is a known limitation of the device, with the manufacturers advising that the MyotonPRO cannot assess the mechanical properties of tissue covered by > 2 cm adipose tissue [28]. Our results, however, indicate that this does not seem to be a specific cutoff point whereafter stiffness cannot be assessed but would rather indicate that any amount of adipose tissue can influence the measurement. This relationship may well be linear in nature as the data would suggest that the influence increases linearly with SAT.

These results are similar to those found by Mencel et al. (2021) [22], who observed a significant negative correlation (*r* = −0.460) between stiffness estimated by the MyotonPRO and skinfold thickness of the RF with participants supine. Our results are further supported by similar findings in the VL muscle, wherein a significant negative correlation (*r* = −0.459, *p* = 0.001) was found between MyotonPRO dynamic stiffness and SAT [23]. Two studies have found significant positive relationships between muscle stiffness assessed by SWE and the MyotonPRO in the lower leg muscles (medial and lateral gastrocnemius and tibialis anterior) [17, 30]. Despite these studies not reporting SAT, it is highly likely that SAT overlying the lower leg muscles in these studies was lower than that overlying the RF and VL of the participants in the present study and the research of Mencel et al. and Bravo‐Sanchez and colleagues. This assumption is based upon previous extensive research into SAT assessment using ultrasound and skinfold measurements [22, 31]. A stronger correlation between the two outcomes would, therefore, be expected as there is a smaller dampening effect on the MyotonPRO's mechanical impulse caused by SAT. These results therefore suggest that, although increasing muscle length improves the agreement between SWE and the MyotonPRO, it is still greatly limited by the disruption of the measurement caused by non‐contractile tissue overlying the targeted muscle. Future research should focus on examining the relationship between SWE, the MyotonPRO, and SAT in other muscles to examine whether there is a greater agreement between SWE and the MyotonPRO in muscles with less overlying noncontractile tissue. Moreover, mechanistic studies utilizing gel phantoms of known stiffness should be used to replicate physiological conditions, similar to those used by Bartsch et al. (2023) [32]. These models are made such that less stiff phantoms (representing SAT) of different thicknesses are placed on top of a stiffer phantom (representing muscle) to examine how this affects dynamic stiffness recordings [19]. This may also allow for the calculation of mathematical corrections to account for participant adiposity, which would enable more accurate utilization of the MyotonPRO.

Since our results in REL imply that RF muscle stiffness measurements using the MyotonPRO do not agree with SWE, it should be considered that they may be measuring two distinct mechanical properties. This is likely due to their individual mechanisms of measurement. Due to the measurement approach of the MyotonPRO, it is conceivable that it is providing an estimation of tissue hardness or compressibility as opposed to stiffness. Hardness can be defined as the resistance of a material to localized plastic deformation, and compressibility can be defined as the change in muscle thickness in response to an external compressive force [33]. Indeed, the MyotonPRO measurement resembles that of a hardness measurement using a durometer with the difference between the two being the dynamic nature of probe compression by the MyotonPRO [34]. Both, however, measure tissue displacement parallel to the external compression force. This is different from SWE which induces shear waves perpendicular to the US transducer orientation, traveling longitudinally along the muscle fascicles. The agreement between SWE and the MyotonPRO in NEU and PAST therefore makes sense, as the two devices are measuring different, but related, mechanical properties which change in the same way as muscle length is increased by knee flexion. This aligns with findings by Kelly and colleagues of agreement between the MyotonPRO and SWE in the infraspinatus, erector spinae, and medial gastrocnemius muscles at rest, 40% and 80% of maximal voluntary isometric contraction (MVIC) [35]. These correlations were found when combining data from rest and the two MVIC intensities, wherein both methods reported greater stiffness during contraction and at the higher MVIC. This does not, however, mean that both methods are accurately estimating muscle stiffness. Both muscle stiffness and hardness are known to increase during contraction and with contraction intensity [36, 37]. It is therefore possible that, in this study, SWE was estimating the increases in muscle stiffness in response to contraction, and the MyotonPRO was estimating the increases in muscle hardness in response to contraction, resulting in a positive correlation between the two methods, despite measuring two distinct mechanical properties [35]. This suggests that in certain conditions SWE and the MyotonPRO may provide similar or different data. This is because they are estimating distinct, but related, mechanical properties. Future research should be conducted which decouples stiffness and hardness, and compares measurements of gel phantoms and human muscle using a durometer and the MyotonPRO to establish exactly what the MyotonPRO is measuring.

There are several limitations to this study. Firstly, although care was taken to ensure there was no compression of the US transducer on the skin (through use of a probe holder and visualization of a thick layer of acoustic coupling gel) it is not possible to confirm that this effect was null. This will have had an impact on both the measurement of SWV and SAT [38]. Secondly, despite participants being asked to remain relaxed during measurements, it was not possible to ensure participants were completely still and that the degree of muscle relaxation was optimized, since we did not measure muscle activity using electromyography. Thirdly, although excellent repeatability was exhibited by the MyotonPRO, the same level of repeatability was not observed for SWV. For intra‐day repeatability, 5/36 measurement locations exhibited poor repeatability, 16/36 moderate repeatability, and 15/36 good repeatability, with none displaying excellent repeatability. For inter‐day repeatability, 1/36 measurement locations exhibited poor repeatability, 11/36 moderate repeatability, 19/36 good repeatability, and 5/36 displayed excellent repeatability. Furthermore, due to the minimum sizing of a ROI being 2.2 × 0.5 cm on our US device, some adjacent tissue was included in the SWV assessment of the SKIN and FAS, and, as such, the measurements are not entirely representative of solely the target tissue. Furthermore, this may have, in some instances where muscle thickness was low, caused a partial overlap in ROI assessment area for SUP and DEEP measurements. Finally, in female participants, we did not account for menstrual cycle phase. Whilst evidence for fluctuations in muscle stiffness across the menstrual cycle is weak, there is some evidence to suggest that it may be higher during the follicular phase than during ovulation, measured by both the MyotonPRO and SWE [39, 40]. More research is required to understand if and how the menstrual cycle affects muscle stiffness, and the interaction of this with exercise. Future research should also establish whether sex differences exist in the relationship between muscle stiffness estimated by SWE and the MyotonPRO.

## 5 Perspectives

This study demonstrated that to estimate RF stiffness using the MyotonPRO more accurately, the muscle should be lengthened in the PAST condition. Furthermore, the MyotonPRO device underestimates RF muscle stiffness as a consequence of increasing SAT. The findings in this study have relevance in the use of the MyotonPRO in both clinical and athletic populations. Our results indicate that it is better utilized in lean subjects or in muscles covered by minimal overlying non‐contractile tissue. It is likely, however, that the MyotonPRO is estimating mechanical hardness and not stiffness. Therefore, mechanistic studies must be conducted, and this study replicated in other muscles to further establish the validity of the MyotonPRO as a method of estimating muscle stiffness.

## Conflicts of Interest

The authors declare no conflicts of interest.

## Supporting information


Data S1.

Data S2.

## Data Availability

The data that support the findings of this study are available from the corresponding author upon reasonable request.

## References

[1] R. L. Lieber and B. I. Binder‐Markey , “Biochemical and Structural Basis of the Passive Mechanical Properties of Whole Skeletal Muscle,” Journal of Physiology 599, no. 16 (2021): 3809–3823, 10.1113/JP280867. 34101193 PMC8364503

[2] R. Kelc , J. Naranda , M. Kuhta , and M. Vogrin , “The Physiology of Sports Injuries and Repair Processes,” in Current Issues in Sports and Exercise Medicine, ed. H. Michael , D. Nick , and K. Yaso (IntechOpen, 2013).

[3] J. T. Kalkhoven and M. L. Watsford , “The Relationship Between Mechanical Stiffness and Athletic Performance Markers in Sub‐Elite Footballers,” Journal of Sports Sciences 36, no. 9 (2018): 1022–1029, 10.1080/02640414.2017.1349921. 28697691

[4] N. Miyamoto , K. Hirata , K. Inoue , and T. Hashimoto , “Muscle Stiffness of the Vastus Lateralis in Sprinters and Long‐Distance Runners,” Medicine and Science in Sports and Exercise 51, no. 10 (2019): 2080–2087, 10.1249/mss.0000000000002024. 31525172

[5] E. C. Pickering Rodriguez , M. L. Watsford , R. G. Bower , and A. J. Murphy , “The Relationship Between Lower Body Stiffness and Injury Incidence in Female Netballers,” Sports Biomechanics 16, no. 3 (2017): 361–373, 10.1080/14763141.2017.1319970. 28553879

[6] M. L. Watsford , A. J. Murphy , K. A. McLachlan , et al., “A Prospective Study of the Relationship Between Lower Body Stiffness and Hamstring Injury in Professional Australian Rules Footballers,” American Journal of Sports Medicine 38, no. 10 (2010): 2058–2064. 20595555 10.1177/0363546510370197

[7] J. E. Brandenburg , S. F. Eby , P. Song , et al., “Quantifying Passive Muscle Stiffness in Children With and Without Cerebral Palsy Using Ultrasound Shear Wave Elastography,” Developmental Medicine and Child Neurology 58, no. 12 (2016): 1288–1294, 10.1111/dmcn.13179. 27374483 PMC5118061

[8] S. S. M. Lee , D. Gaebler‐Spira , L.‐Q. Zhang , W. Z. Rymer , and K. M. Steele , “Use of Shear Wave Ultrasound Elastography to Quantify Muscle Properties in Cerebral Palsy,” Clinical Biomechanics 31 (2016): 20–28, 10.1016/j.clinbiomech.2015.10.006. 26490641 PMC4729598

[9] J. Marusiak , K. Kisiel‐Sajewicz , A. Jaskólska , and A. Jaskólski , “Higher Muscle Passive Stiffness in Parkinson's Disease Patients Than in Controls Measured by Myotonometry,” Archives of Physical Medicine and Rehabilitation 91, no. 5 (2010): 800–802, 10.1016/j.apmr.2010.01.012. 20434620

[10] T. Kodesho , K. Taniguchi , T. Kato , et al., “Relationship Between Shear Elastic Modulus and Passive Force of the Human Rectus Femoris at Multiple Sites: A Thiel Soft‐Embalmed Cadaver Study,” Journal of Medical Ultrasonics 48, no. 2 (2021): 115–121, 10.1007/s10396-020-01076-w. 33576917

[11] G. Nakao , T. Kodesho , T. Kato , et al., “Relationship Between Shear Elastic Modulus and Passive Muscle Force in Human Hamstring Muscles Using a Thiel Soft‐Embalmed Cadaver,” Journal of Medical Ultrasonics 50 (2023): 275–283, 10.1007/s10396-023-01317-8. 37170041 PMC10954965

[12] L. C. Davis , T. G. Baumer , M. J. Bey , and M. Van Holsbeeck , “Clinical Utilization of Shear Wave Elastography in the Musculoskeletal System,” Ultrasonography 38, no. 1 (2019): 2–12. 30343557 10.14366/usg.18039 PMC6323314

[13] N. Frulio and H. Trillaud , “Ultrasound Elastography in Liver. Diagnostic and Interventional,” Imaging 94, no. 5 (2013): 515–534, 10.1016/j.diii.2013.02.005. 23623211

[14] D. O. Cosgrove , W. A. Berg , C. J. Doré , et al., “Shear Wave Elastography for Breast Masses Is Highly Reproducible,” European Radiology 22, no. 5 (2012): 1023–1032, 10.1007/s00330-011-2340-y. 22210408 PMC3321140

[15] L. Lacourpaille , A. Nordez , F. Hug , V. Doguet , R. Andrade , and G. Guilhem , “Early Detection of Exercise‐Induced Muscle Damage Using Elastography,” European Journal of Applied Physiology 117, no. 10 (2017): 2047–2056, 10.1007/s00421-017-3695-9. 28780603

[16] G. Guilhem , V. Doguet , H. Hauraix , et al., “Muscle Force Loss and Soreness Subsequent to Maximal Eccentric Contractions Depend on the Amount of Fascicle Strain In Vivo,” Acta Physiologica 217, no. 2 (2016): 152–163. 26786411 10.1111/apha.12654

[17] Y. Feng , Y. Li , C. Liu , and Z. Zhang , “Assessing the Elastic Properties of Skeletal Muscle and Tendon Using Shearwave Ultrasound Elastography and MyotonPRO,” Scientific Reports 8, no. 1 (2018): 1–9. 30459432 10.1038/s41598-018-34719-7 PMC6244233

[18] “Myoton,” accessed 12/08/2024, https://myoton.com/technology/.

[19] W. Kurashina , Y. Iijima , H. Sasanuma , T. Saito , and K. Takeshita , “Evaluation of Muscle Stiffness in Adhesive Capsulitis With Myoton PRO,” JSES International 7, no. 1 (2023): 25–29, 10.1016/j.jseint.2022.08.017. 36820433 PMC9937826

[20] Y. Lee , M. Kim , and H. Lee , “The Measurement of Stiffness for Major Muscles With Shear Wave Elastography and Myoton: A Quantitative Analysis Study,” Diagnostics 11, no. 3 (2021): 524. 33804273 10.3390/diagnostics11030524 PMC7999852

[21] A. Kawczyński , D. Mroczek , R. E. Andersen , T. Stefaniak , L. Arendt‐Nielsen , and P. Madeleine , “Trapezius Viscoelastic Properties Are Heterogeneously Affected by Eccentric Exercise,” Journal of Science and Medicine in Sport 21, no. 8 (2018): 864–869, 10.1016/j.jsams.2018.01.005. 29395631

[22] J. Mencel , A. Jaskólska , J. Marusiak , et al., “Effect of Gender, Muscle Type and Skinfold Thickness on Myometric Parameters in Young People,” PeerJ 9 (2021): e12367. 34824907 10.7717/peerj.12367 PMC8590390

[23] A. Bravo‐Sánchez , P. Abián , J. Sánchez‐Infante , P. Esteban‐Gacía , F. Jiménez , and J. Abián‐Vicén , “Objective Assessment of Regional Stiffness in Vastus Lateralis With Different Measurement Methods: A Reliability Study,” Sensors 21, no. 9 (2021): 3213. 34066343 10.3390/s21093213 PMC8125613

[24] S. Taş and Y. Salkın , “An Investigation of the Sex‐Related Differences in the Stiffness of the Achilles Tendon and Gastrocnemius Muscle: Inter‐Observer Reliability and Inter‐Day Repeatability and the Effect of Ankle Joint Motion,” Foot 41 (2019): 44–50, 10.1016/j.foot.2019.09.003. 31704588

[25] J. Zhou , Y. Lin , J. Zhang , et al., “Reliability of Shear Wave Elastography for the Assessment of Gastrocnemius Fascia Elasticity in Healthy Individual,” Scientific Reports 12, no. 1 (2022): 8698, 10.1038/s41598-022-12786-1. 35610329 PMC9130247

[26] P. S. Lall , A. M. Alsubiheen , M. M. Aldaihan , and H. Lee , “Differences in Medial and Lateral Gastrocnemius Stiffness After Exercise‐Induced Muscle Fatigue,” International Journal of Environmental Research and Public Health 19, no. 21 (2022): 13891, 10.3390/ijerph192113891. 36360770 PMC9656849

[27] T. K. Koo and M. Y. Li , “A Guideline of Selecting and Reporting Intraclass Correlation Coefficients for Reliability Research,” Journal of Chiropractic Medicine 15, no. 2 (2016): 155–163, 10.1016/j.jcm.2016.02.012. 27330520 PMC4913118

[28] “Myoton,” https://www.myoton.com/UserFiles/Updates/MyotonPRO_User_Manual.pdf.

[29] P. Schober , C. Boer , and L. A. Schwarte , “Correlation Coefficients: Appropriate Use and Interpretation,” Anesthesia and Analgesia 126, no. 5 (2018): 1763–1768. 29481436 10.1213/ANE.0000000000002864

[30] Y. Do , P. S. Lall , and H. Lee , “Assessing the Effects of Aging on Muscle Stiffness Using Shear Wave Elastography and Myotonometer,” Health 9, no. 12 (2021): 1733, 10.3390/healthcare9121733. PMC8700831 34946459

[31] W. Müller , A. Fürhapter‐Rieger , H. Ahammer , et al., “Relative Body Weight and Standardised Brightness‐Mode Ultrasound Measurement of Subcutaneous Fat in Athletes: An International Multicentre Reliability Study, Under the Auspices of the IOC Medical Commission,” Sports Medicine 50, no. 3 (2020): 597–614, 10.1007/s40279-019-01192-9. 31571156 PMC7018793

[32] K. Bartsch , A. Brandl , P. Weber , et al., “Assessing Reliability and Validity of Different Stiffness Measurement Tools on a Multi‐Layered Phantom Tissue Model,” Scientific Reports 13, no. 1 (2023): 815, 10.1038/s41598-023-27742-w. 36646734 PMC9842673

[33] D. Parker and F. Hashmi , “Chapter 15 ‐ Application of Tissue Mechanics to Clinical Management of Risk in the Diabetic Foot,” in The Science, Etiology and Mechanobiology of Diabetes and Its Complications, ed. A. Gefen (Academic Press, 2021), 255–281.

[34] M. Helili , X. Geng , X. Ma , et al., “An Investigation of Regional Plantar Soft Tissue Hardness and Its Potential Correlation With Plantar Pressure Distribution in Healthy Adults,” Applied Bionics and Biomechanics 2021 (2021): 5566036, 10.1155/2021/5566036. 34239603 PMC8241530

[35] J. P. Kelly , S. L. Koppenhaver , L. A. Michener , L. Proulx , F. Bisagni , and J. A. Cleland , “Characterization of Tissue Stiffness of the Infraspinatus, Erector Spinae, and Gastrocnemius Muscle Using Ultrasound Shear Wave Elastography and Superficial Mechanical Deformation,” Journal of Electromyography and Kinesiology 38 (2018): 73–80. 29175615 10.1016/j.jelekin.2017.11.001

[36] Y. Yoshitake , Y. Takai , H. Kanehisa , and M. Shinohara , “Muscle Shear Modulus Measured With Ultrasound Shear‐Wave Elastography Across a Wide Range of Contraction Intensity,” Muscle & Nerve 50, no. 1 (2014): 103–113, 10.1002/mus.24104. 24155045

[37] T. Inami , T. Tsujimura , T. Shimizu , T. Watanabe , W. Y. Lau , and K. Nosaka , “Relationship Between Isometric Contraction Intensity and Muscle Hardness Assessed by Ultrasound Strain Elastography,” European Journal of Applied Physiology 117, no. 5 (2017): 843–852, 10.1007/s00421-016-3528-2. 28290056

[38] J. Vachutka , Z. Sedlackova , T. Furst , et al., “Evaluation of the Effect of Tissue Compression on the Results of Shear Wave Elastography Measurements,” Ultrasonic Imaging 40, no. 6 (2018): 380–393, 10.1177/0161734618793837. 30101677

[39] J. Saeki , T. Ikezoe , S. Yoshimi , M. Nakamura , and N. Ichihashi , “Menstrual Cycle Variation and Gender Difference in Muscle Stiffness of Triceps Surae,” Clinical Biomechanics 61 (2019): 222–226, 10.1016/j.clinbiomech.2018.12.013. 30599387

[40] I. A. Khowailed , Y. Lee , and H. Lee , “Assessing the Differences in Muscle Stiffness Measured With Shear Wave Elastography and Myotonometer During the Menstrual Cycle in Young Women,” Clinical Physiology and Functional Imaging 42, no. 5 (2022): 320–326, 10.1111/cpf.12763. 35596621

