# Supplementary material for: MyotonPRO Is Not Comparable to Shear Wave Elastography in the Measurement of Rectus Femoris Muscle Stiffness due to Interference of Subcutaneous Adipose Tissue
Source: Scand J Med Sci Sports. 2025 Jul 25;35(8):e70095. doi: 10.1111/sms.70095 (PMC12291615; doi:10.1111/sms.70095)
Supplement: Supplementary file 1 — Data S1. [file SMS-35-e70095-s002.docx]

| Condition | Region | Depth | ICC (single measures) | Lower bound | Upper bound | Standard error of measurement (m/s) |
| --- | --- | --- | --- | --- | --- | --- |
| Relaxed | Proximal | Skin | 0.900 | 0.671 | 0.965 | 0.141 |
|  |  | Fascia | 0.883 | 0.669 | 0.956 | 0.184 |
|  |  | Superficial muscle | 0.743 | 0.397 | 0.896 | 0.219 |
|  |  | Deep muscle | 0.740 | 0.453 | 0.888 | 0.232 |
|  | Medial | Skin | 0.829 | 0.621 | 0.928 | 0.158 |
|  |  | Fascia | 0.854 | 0.670 | 0.939 | 0.122 |
|  |  | Superficial muscle | 0.711 | 0.398 | 0.875 | 0.138 |
|  |  | Deep muscle | 0.538 | 0.126 | 0.789 | 0.145 |
|  | Distal | Skin | 0.686 | 0.370 | 0.861 | 0.138 |
|  |  | Fascia | 0.743 | 0.452 | 0.890 | 0.110 |
|  |  | Superficial muscle | 0.886 | 0.736 | 0.953 | 0.055 |
|  |  | Deep muscle | 0.474 | 0.077 | 0.748 | 0.134 |
| Neutral | Proximal | Skin | 0.788 | 0.545 | 0.910 | 0.100 |
|  |  | Fascia | 0.934 | 0.841 | 0.973 | 0.110 |
|  |  | Superficial muscle | 0.653 | 0.315 | 0.846 | 0.170 |
|  |  | Deep muscle | 0.904 | 0.772 | 0.961 | 0.130 |
|  | Medial | Skin | 0.882 | 0.724 | 0.951 | 0.122 |
|  |  | Fascia | 0.686 | 0.364 | 0.863 | 0.214 |
|  |  | Superficial muscle | 0.838 | 0.634 | 0.933 | 0.145 |
|  |  | Deep muscle | 0.864 | 0.691 | 0.944 | 0.170 |
|  | Distal | Skin | 0.907 | 0.770 | 0.963 | 0.161 |
|  |  | Fascia | 0.894 | 0.751 | 0.957 | 0.122 |
|  |  | Superficial muscle | 0.662 | 0.315 | 0.852 | 0.179 |
|  |  | Deep muscle | 0.762 | 0.497 | 0.898 | 0.164 |
| Passively stretched | Proximal | Skin | 0.834 | 0.633 | 0.930 | 0.155 |
|  |  | Fascia | 0.837 | 0.633 | 0.932 | 0.270 |
|  |  | Superficial muscle | 0.907 | 0.781 | 0.962 | 0.155 |
|  |  | Deep muscle | 0.872 | 0.709 | 0.947 | 0.200 |
|  | Medial | Skin | 0.844 | 0.651 | 0.935 | 0.190 |
|  |  | Fascia | 0.912 | 0.792 | 0.964 | 0.122 |
|  |  | Superficial muscle | 0.733 | 0.439 | 0.885 | 0.197 |
|  |  | Deep muscle | 0.854 | 0.668 | 0.939 | 0.200 |
|  | Distal | Skin | 0.748 | 0.466 | 0.892 | 0.263 |
|  |  | Fascia | 0.864 | 0.686 | 0.944 | 0.253 |
|  |  | Superficial muscle | 0.815 | 0.588 | 0.923 | 0.235 |
|  |  | Deep muscle | 0.945 | 0.868 | 0.978 | 0.158 |

**Table 1**: Reproducibility results for shear wave elastography in relaxed, neutral and passively stretched conditions; proximal, medial and distal, regions; and skin, fascia, superficial muscle and deep muscle. Measures of relative and absolute repeatability are presented as intraclass correlation coefficient (ICC) and standard error of the measurement, respectively.
