# Supplementary material for: MyotonPRO Is Not Comparable to Shear Wave Elastography in the Measurement of Rectus Femoris Muscle Stiffness due to Interference of Subcutaneous Adipose Tissue
Source: Scand J Med Sci Sports. 2025 Jul 25;35(8):e70095. doi: 10.1111/sms.70095 (PMC12291615; doi:10.1111/sms.70095)
Supplement: Supplementary file 2 — Data S2. [file SMS-35-e70095-s001.docx]

| Measurement location | | | Pearson correlation | | Simple linear regression | | |
| --- | --- | --- | --- | --- | --- | --- | --- |
| Condition | Region | Depth | R value | P value | R^2^ value | Lower bound | Upper bound |
| Relaxed | Proximal | Skin | 0.183 | 0.226 | 0.034 | -24.244 | 52.046 |
|  |  | Fascia | 0.347 | 0.073 | 0.120 | -8.714 | 54.243 |
|  |  | Superficial muscle | 0.474 | **0.020** | 0.225 | 1.980 | 78.065 |
|  |  | Deep muscle | 0.169 | 0.245 | 0.029 | -30.471 | 61.152 |
|  | Medial | Skin | -0.050 | 0.418 | 0.002 | -38.633 | 31.573 |
|  |  | Fascia | 0.091 | 0.351 | 0.008 | -34.584 | 50.323 |
|  |  | Superficial muscle | 0.061 | 0.399 | 0.004 | -48.755 | 62.550 |
|  |  | Deep muscle | 0.097 | 0.342 | 0.009 | -55.032 | 81.988 |
|  | Distal | Skin | 0.009 | 0.485 | 0.000 | -86.183 | 89.403 |
|  |  | Fascia | 0.290 | 0.107 | 0.084 | -37.859 | 157.635 |
|  |  | Superficial muscle | -0.022 | 0.463 | 0.000 | -144.615 | 132.365 |
|  |  | Deep muscle | 0.104 | 0.332 | 0.011 | -97.821 | 149.984 |
|  | Pooled | Skin | -0.023 | 0.430 | 0.001 | -29.044 | 24.351 |
|  |  | Fascia | -0.214 | 0.052 | 0.046 | -29.314 | 2.776 |
|  |  | Superficial muscle | -0.182 | 0.084 | 0.033 | -38.450 | 6.888 |
|  |  | Deep muscle | -0.267 | **0.020** | 0.071 | -48.049 | -1.055 |
| Neutral | Proximal | Skin | -0.321 | 0.090 | 0.103 | -105.955 | 21.585 |
|  |  | Fascia | 0.060 | 0.403 | 0.004 | -30.700 | 38.919 |
|  |  | Superficial muscle | 0.064 | 0.397 | 0.004 | -47.321 | 60.928 |
|  |  | Deep muscle | 0.317 | 0.093 | 0.101 | -11.694 | 55.952 |
|  | Medial | Skin | 0.300 | 0.099 | 0.090 | -18.502 | 82.853 |
|  |  | Fascia | 0.301 | 0.099 | 0.090 | -19.194 | 86.449 |
|  |  | Superficial muscle | 0.240 | 0.154 | 0.058 | -27.927 | 83.838 |
|  |  | Deep muscle | 0.364 | 0.057 | 0.133 | -8.588 | 73.013 |
|  | Distal | Skin | 0.139 | 0.280 | 0.019 | -34.032 | 60.937 |
|  |  | Fascia | 0.713 | **<.001** | 0.508 | 54.048 | 156.799 |
|  |  | Superficial muscle | 0.564 | **0.005** | 0.318 | 30.145 | 189.003 |
|  |  | Deep muscle | 0.510 | **0.011** | 0.260 | 14.118 | 157.116 |
|  | Pooled | Skin | 0.662 | **<.001** | 0.438 | 62.955 | 117.016 |
|  |  | Fascia | 0.262 | **0.023** | 0.069 | 1.170 | 105.190 |
|  |  | Superficial muscle | 0.236 | **0.036** | 0.055 | -5.669 | 125.857 |
|  |  | Deep muscle | 0.315 | **0.008** | 0.099 | 12.333 | 110.156 |
| Passively stretched | Proximal | Skin | 0.243 | 0.158 | 0.059 | -33.309 | 97.352 |
|  |  | Fascia | 0.176 | 0.235 | 0.031 | -24.814 | 51.511 |
|  |  | Superficial muscle | 0.309 | 0.099 | 0.095 | -17.295 | 77.353 |
|  |  | Deep muscle | 0.555 | **0.007** | 0.308 | 11.626 | 87.839 |
|  | Medial | Skin | 0.252 | 0.142 | 0.063 | -22.334 | 71.721 |
|  |  | Fascia | 0.232 | 0.162 | 0.054 | -27.962 | 79.986 |
|  |  | Superficial muscle | 0.318 | 0.086 | 0.101 | -19.545 | 101.961 |
|  |  | Deep muscle | 0.623 | **0.002** | 0.388 | 21.327 | 91.507 |
|  | Distal | Skin | 0.423 | **0.032** | 0.179 | -2.796 | 94.373 |
|  |  | Fascia | 0.774 | **<.001** | 0.600 | 37.227 | 87.825 |
|  |  | Superficial muscle | 0.640 | **0.001** | 0.410 | 26.882 | 105.493 |
|  |  | Deep muscle | 0.695 | **<.001** | 0.482 | 27.270 | 84.696 |
|  | Pooled | Skin | 0.600 | **<.001** | 0.359 | 45.804 | 96.023 |
|  |  | Fascia | 0.196 | 0.069 | 0.038 | -7.271 | 51.419 |
|  |  | Superficial muscle | 0.261 | **0.023** | 0.068 | 0.702 | 74.280 |
|  |  | Deep muscle | 0.454 | **<.001** | 0.206 | 25.096 | 79.722 |

**Table 2**: Results from Pearson’s correlations, and simple linear regressions between shear wave velocity and dynamic stiffness, measured by the MyotonPRO, in relaxed, neutral and passively stretched conditions; proximal, medial and distal, and pooled, regions; and skin, fascia, superficial muscle and deep muscle.
